# Breastfeeding Measurement—What Does It Mean to “Wean?”

**DOI:** 10.1177/08903344251320034

**Published:** 2025-02-24

**Authors:** Melissa A. Theurich, Laura Fischer, Jelica Gencel-Augusto, Ellen Chetwynd

**Affiliations:** 1 Chair of Public Health and Health Services Research, Ludwig-Maximilians-Universität München (LMU Munich), Munich, Germany; 2 Pettenkofer School of Public Health, Munich, Germany; 3 School of Medicine, Department of Family Medicine, University of North Carolina at Chapel Hill, Chapel Hill, NC, USA

**Keywords:** breastfeeding, breastfeeding assessment, breastfeeding rates, complementary feeding, weaning

## Abstract

The term “weaning” is used heterogeneously in scientific and gray literature, with no commonly agreed-upon definition. Weaning can describe the gradual transition in the infant diet, usually from milk feedings to complementary foods, but it is also commonly used to describe any gradual transition between milks, foods, or feeding modalities. In an epidemiological context, it may also refer to changing breastfeeding rates within a cohort. The meaning of weaning has changed over time and may vary by the scientific assumptions applied to this period of human development. For these reasons, we propose that investigators avoid using the term weaning, and substitute it with more precise terminology. We present a series of proposed standard terms with corresponding definitions to guide more precise and accurate reporting of the various potential meanings of the term in the scientific literature. The objective is to improve reporting and reproducibility of research in the field of breastfeeding and human lactation.

## Introduction

For the last decade or more, there has been a rising appreciation of the need for consistent definitions of breastfeeding in research (Labbok & Starling, 2012). This article expands on the concepts of “lactation and breastfeeding terminology for population health research” as recently outlined (Yourkavitch & Chetwynd, 2019). To our knowledge, no commonly agreed definition for weaning has been established. This article addresses the variability in terminology around weaning, exploring key considerations for defining and reporting it. Our objective is to guide researchers to use standardized terms in order to more precisely and accurately describe dietary changes taking place during the first years of life.

## Historical Considerations

Records of the term “weaning” date back at least to the 1800s (Mayes, 1846). Historically, the term has been used to describe the process of transitioning from breastmilk as the sole source of child nutrition, to complete reliance on other foods—most commonly, animal milk (Burnet, 1904; Crouch, 1898; McHatton, 1908). In the 1980s, for example, the World Health Organization (WHO) referred to complementary feeding as weaning and referred to complementary foods as weaning foods (WHO, 1988). In the 1990s, Greiner addressed the scientific community, urging them to avoid the term weaning and to rather specify the nature of the changes in the diet (Greiner, 1996). However, up until today, weaning continues to be used in a heterogeneous manner in scientific literature and national guidelines. For example, in the United Kingdom, it is used synonymously with “complementary feeding” (British Dietetic Association, 2020; National Health Service, 2022, 2025), while the United States Department of Agriculture (USDA) uses the term complementary feeding and does not refer to weaning (U.S. Department of Agriculture & U.S. Department of Health and Human Services, December 2020). The WHO has largely stopped referring to complementary feeding as weaning since the latter implies a reduction, replacement, or cessation of breastfeeding, which would contradict global recommendations that encourage continued breastfeeding during the second half of the first year of life alongside complementary foods. The most recent WHO guideline for complementary feeding of infants and young children 6–23 months of age no longer uses the word weaning to refer to complementary feeding (WHO, 2023).

### The Multifaceted Definitions of Weaning

The term weaning is commonly used in the context of complementary feeding to refer to the nutritional transition from a liquid milk diet to the family diet. However, it can also refer to a reduction in breastfeeding (or expressed human milk feedings) over a specified period of time or a reduction in the overall volume of human milk feedings. In clinical practice, weaning might refer to a transition in feeding schedules (e.g., reducing only nighttime or only daytime feedings), which might occur when a child is being taken care of by someone outside of the home.

In scientific research, the term weaning may refer to a time period. For example, in epidemiological studies, researchers might measure the time until complete breastfeeding cessation or the time until the introduction of other foods. In clinical lactation and medical research, weaning may also refer to a transition between feeding modalities (e.g., being weaned from an infant feeding bottle to direct breastfeeding). The following sections detail the most common meanings of weaning. Table 1 provides recommendations for standardized terminology to improve the scientific accuracy and reproducibility of studies investigating this time period.

**Table 1.** Proposed Terminology With Corresponding Definitions.

Proposed Term	Proposed Definition
Complementary Feeding	We propose to use the WHO definition of complementary feeding: “The process of providing foods in addition to milk when breast milk or milk formula alone are no longer adequate to meet nutritional requirements” (WHO, 2023).
Cessation of Direct Breastfeeding	We propose to define “cessation of direct breastfeeding” as stopping putting a child to the breast (including cessation of wet nursing). In transgender or non-gender conforming individuals, terminology might be amended as preferred, e.g., cessation of direct “chestfeeding.”
Cessation of Any Human Milk Feeding	We propose to define “cessation of any human milk feeding” as ending the provision of any form of human milk (e.g., expressed, fresh, frozen, etc.).
Breastfeeding Attrition	A survival analysis (time-to-event analysis) is used in epidemiological studies to analyze the time until an event (i.e., cessation of breastfeeding or cessation of exclusive breastfeeding) occurs within a cohort. We propose to define “breastfeeding attrition” as a rate, calculated by dividing the number of breastfeeding dyads who stopped (exclusively) breastfeeding over a time period by the average number of breastfeeding dyads observed in that time period.
Transitions in Parenteral or Enteral Nutrition	When referring to transitions between the content of the nutritional support provided, either enterally (such as milks or formulas through the digestive tract) or parenterally (such as special nutritional preparations through venous access), we propose to specify both the nature of the nutritional support being replaced and the new support being provided.
Transition in Feeding Modalities	In medical and clinical lactation studies, when referring to a transition in feeding modalities, we propose to specify which feeding modalities are being replaced by which other feeding modalities.

## Definition 1: Complementary Feeding

**Figure 1.**
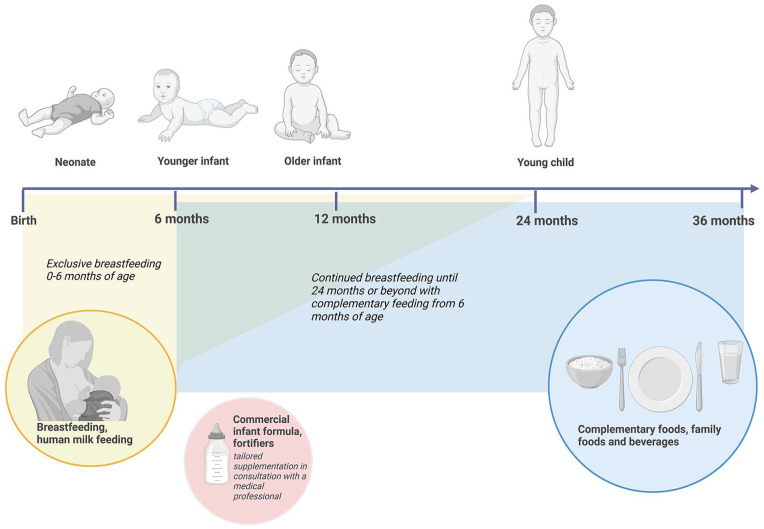
Global Recommendations for Infant and Young Child Feeding (0–36 Months). *Note*: WHO and UNICEF recommend that children initiate breastfeeding within the first hour of birth and be exclusively breastfed for the first 6 months of life. From the age of 6 months, children should begin eating safe and adequate complementary foods while continuing to breastfeed for up to two years of age or beyond.

The term weaning has traditionally been used to refer to the time period around the middle of the first year of life when breastfed infants slowly and gradually transition from a diet of exclusive human milk to a diet of animal and plant-based complementary foods. This period is synonymously called the “complementary feeding period.” The WHO recommends exclusive breastfeeding for the first 6 months of life and continued breastfeeding with complementary foods until 2 years of age or beyond. In mixed-fed or commercial infant formula-fed infants, the complementary feeding period refers to the gradual transition from any milks (human, animal, commercial infant formula) to complementary foods. Figure 1 depicts human development between birth and 36 months of age and highlights the corresponding gradual dietary transition from exclusive human milk feeding to the family diet. In the context of the gradual provision of complementary foods to the infant diet, we propose to use the term “complementary feeding” instead of the term “weaning.”

**Key Messages**

- There are inconsistencies in the terminology used around “weaning” in scientific literature, as well as in national and international white papers.
- Use of the term weaning has changed over time, may have different meanings based on geography, and may vary by scientific assumptions that surround this period of the human lifespan.
- Unstandardized definitions of weaning may limit the interpretation and usefulness of breastfeeding, lactation, and early childhood nutrition research.
- Researchers should avoid using the term weaning or alternatively aim to clearly define “weaning” in order to improve scientific accuracy and reproducibility.

### Definition 2: Breastfeeding Cessation

The verb “to wean” may also refer to the complete cessation of breastfeeding, for example, a mother might report, “I weaned my child over the weekend.” Most typically, this implies that the child was no longer directly breastfeeding . Given the common practice in some countries of expressing and storing human milk, it is possible that a child who has ceased direct breastfeeding could still be receiving stored human milk. Infants may also receive human milk from a milk bank or from other breastfeeding parents (e.g., informally shared milk). Scientifically, it is important to disentangle terminology around breastfeeding cessation. Therefore, we propose to use the term “cessation of any human milk feeding” as the umbrella term when both direct breastfeeding and the feeding of any and all other sources of human milk have ceased.

In conclusion, in the context of breastfeeding cessation, we recommend terminology referring to the “cessation of direct breastfeeding,” and/or “cessation of any human milk feeding” instead of the term “weaning.” In transgender or non-gender conforming individuals, terminology might be amended as preferred, for example, “cessation of direct chestfeeding.”

### Definition 3: Breastfeeding Attrition

**Figure 2.**
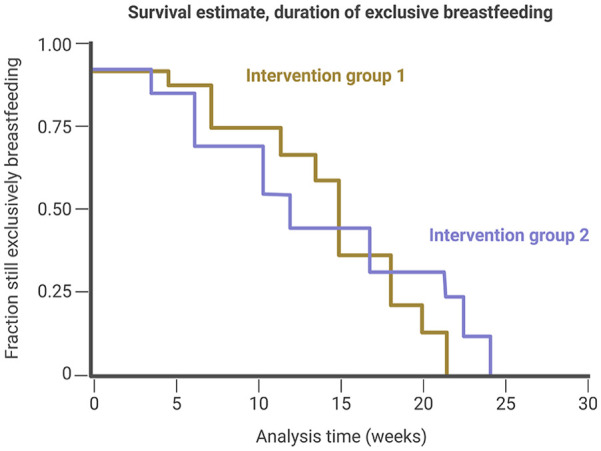
Survival Analysis (Kaplan–Meier) Showing “Breastfeeding Attrition” in a Cohort. *Note.* This figure illustrates a hypothetical representation of “breastfeeding attrition,” with the defined endpoint of “exclusive breastfeeding.” The concept of breastfeeding attrition is not limited to the evaluation of exclusive breastfeeding as the only outcome of interest; therefore, researchers should clearly define their own outcome of interest.

Survival analysis (also known as time-to-event analysis) is a branch of statistics used to analyze the expected duration of time until an event occurs. In breastfeeding and lactation research, it is important for researchers to define the event of interest clearly. For example, the endpoint of interest might either refer to the time until the complete cessation of direct breastfeeding, the time until the complete cessation of any human milk feeding, or the time until the end of “exclusive breastfeeding” (for example, when commercial infant formula or complementary foods are introduced). When describing the number of participants in a cohort that changes from, for example, “(exclusively) breastfeeding” to “not (exclusively) breastfeeding” over time, the term “breastfeeding attrition” can be used (see Figure 2.

The breastfeeding attrition rate is calculated as follows:

$$\frac{\begin{array}{c} Number\;of\;(exclusively)\;breastfeeding\;\\ dyads\;who\;stopped\;breastfeeding\\ \;over\;a\;time\;period \end{array}}{\begin{array}{l} Average\;number\;of\;(exclusively)\;\\ breastfeeding\;dyads\;in\;that\;time\;period \end{array}}=Breastfeeding\;Attrition\;Rate$$

In the context of epidemiological studies investigating the change of breastfeeding rates in a cohort over time, we recommend that researchers clearly define the end point of interest (e.g., cessation of direct breastfeeding, cessation of any human milk feeding, cessation of exclusive breastfeeding) and to use the term breastfeeding attrition instead of the term weaning.

### Definition 4: Transition Between Types of Milks or Other Fluids

Infancy is a period of rapid dietary transitions, where any shift involving the gradual replacement of one source of nutrients with another has been colloquially and clinically described as weaning. This includes transitions between different types of milk, such as human milk, commercial infant formula milks (including changes within commercial infant formula types), and animal milks. The following section offers global definitions for the terms: “breastmilk substitutes,” “commercial milk formulas,” and “animal milks” (see Box 1).

**Box 1.** Types of Human Milk, Animal Milk, Commercial Milk Formula, Water and Beverages.

• **Human milk**, e.g. mother’s own milk, donor milk, human milk from informal milk sharing, milk from a wet nurse, human milk-based fortifiers • **Commercial milk formulas**, e.g. cow’s milk infant formula, plant-based infant formulas, commercial infant formula fortifiers, specially formulated commercial infant formulas for specific disease states, follow-on/follow-up commercial infant formulas, toddler milks • **Synthetic milks**, also known as “lab grown” or “cell-based” refer to milks produced from human or animal cells in a laboratory • **Animal milks**, e.g. unmodified cow, goat, or camel milks • **Water and beverages**, e.g. sweetened and unsweetened water, tea, and other beverages

In the context of the gradual transition between types of milks or other fluids, it is important to specify which nutrient sources are introduced or replaced in the diet, instead of generally referring to weaning.

#### Breastmilk substitutes

The WHO recommends exclusive breastfeeding from birth until 6 months of age. Any commercial infant formula, foods, and beverages offered prematurely, used as a partial or total replacement of breastmilk, are considered breastmilk substitutes.

#### Commercial infant formulas

The Codex Alimentarius Commission (CAC), is the central part of the joint Food and Agriculture Organization (FAO) and WHO Food Standards Program, which was established to protect consumer health and promote fair practices in food trade (WHO & FAO, 2025). The CAC defines commercial infant formulas as “a breast-milk substitute specially manufactured to satisfy, by itself, the nutritional requirements of infants during the first months of life up to the introduction of appropriate complementary feeding” (WHO, 2023). The CAC defines “follow-up”/ “follow-on formula” for older infants as follows: “follow-up formula for older infants means a product, manufactured for use as a breastmilk-substitute, as a liquid part of a diet for older infants when progressively diversified complementary feeding is introduced” (WHO & FAO, 2023).

#### Animal milks

The WHO defines animal milks as “milks from any animal, such as a cow, goat, or camel” (WHO, 2023). According to the WHO, animal milks are considered breastmilk substitutes (and not complementary foods).

### Definition 5: Transition Between Feeding Modalities

A large number of feeding modalities have been described in scientific literature (see Box 2; Theurich et al., 2021). Feeding modalities describe *how* infants are fed.

**Box 2.** Feeding Modalities

• **Direct breastfeeding**, by the mother, through wet nursing and/or aided (e.g., with a lactation aid or nursing supplementer). This may be referred to as chest feeding in transgender individuals. • **Feeding from a device**, such as a bottle, teat, finger, cup, spoon or paladai • **Medical feeding interventions**, such as tube or gavage feeding, or oropharyngeal colostrum administration, and parenteral nutrition

*Note.* Adapted from Theurich et al., 2021.

Infants can be transitioned from any one feeding modality to another. For example, an infant may be transitioned from feeding directly at the breast to feeding from an infant feeding bottle. Therefore, the gradual transition between feeding modalities, while an infant is being accustomed to an alternative feeding mode, is another potential meaning of the term weaning. In the context of the gradual transition between feeding modalities, it is important to specify the modalities, for example, “transition from breast to bottle” or “transition from tube feeding to breast,” instead of generally using the term weaning.

## Conclusion

To date, no commonly agreed definition for weaning has been established. We summarize five ways the term weaning is being used colloquially, clinically, and in scientific and grey literature. Due to the various potential definitions identified, we propose that researchers avoid using the term weaning, and instead use the standard terms and definitions proposed here. The objective of the proposed terminology and corresponding definitions is to improve accuracy and precision of terminology in breastfeeding and human lactation research.

**Table:** 

Key Recommendations 1) In the context of the gradual provision of complementary foods, use the term “complementary feeding” instead of the term “weaning.” 2) In the context of breastfeeding cessation, use the terms “cessation of direct breastfeeding” or “cessation of any human milk” feeding instead of the term “weaning.” 3) In the context of epidemiological studies investigating changes in breastfeeding rates in a cohort, clearly define the end point of interest and use the term “breastfeeding attrition” instead of the term “weaning.” 4) In the context of the gradual transition between types of milks or infant formulas, specify which nutrient sources are introduced or replaced in the diet, instead of generally referring to weaning. 5) In the context of the gradual transition between feeding modalities, specify both modalities, for example, “transition from breast to bottle,” instead of generally referring to weaning.
